# Molecular Characterization and Marker Development of the *HMW-GS* Gene from *Thinopyrum elongatum* for Improving Wheat Quality

**DOI:** 10.3390/ijms241311072

**Published:** 2023-07-04

**Authors:** Yi Dai, Jinfeng Li, Juntao Shi, Yujiao Gao, Haigang Ma, Yonggang Wang, Hongxiang Ma

**Affiliations:** Joint International Research Laboratory of Agriculture and Agri-Product Safety, The Ministry of Education of China/Jiangsu Key Laboratory of Crop Genomics and Molecular Breeding/Jiangsu Co-Innovation Center of Modern Production Technology of Grain Crops, Yangzhou University, Yangzhou 225009, China; daiyi@yzu.edu.cn (Y.D.); mz120201534@stu.yzu.edu.cn (J.L.); sjt0602@163.com (J.S.); yujiaogao@yzu.edu.cn (Y.G.); mhg@yzu.edu.cn (H.M.); yg.wang@yzu.edu.cn (Y.W.)

**Keywords:** wheat relatives, high-molecular-weight glutenin subunit, quality, molecular marker

## Abstract

The quality of wheat primarily depends on its storage protein quality, especially in regards to gluten content and high-molecular-weight glutenin subunits (HMW-GS). The number of HMW-GS alleles is limited in bread wheat (*Triticum aestivum* L.), whereas it is abundant in wheat relatives. Therefore, HMW-GS alleles from wheat relatives could provide a potential for improving quality in wheat breeding. *Thinopyrum elongatum* (EE) is one of the relatives of wheat. The E genome is closely related to the ABD genome in wheat; therefore, *Th. elongatum* is often used as an excellent exogenous gene donor for wheat genetic improvement. In this study, the high-molecular glutenin subunit gene was cloned and sequenced from *Th. elongatum*. A specific molecular marker for identifying the *Glu-1Ey* subunit gene was developed and applied to detected wheat-*Th. elongatum* alien introgression lines. Quality analysis indicated that the substitution and addition lines containing *Th. elongatum* alleles significantly (*p* < 0.05) increased grain protein content by 3.76% to 5.11%, wet-gluten content by 6.55% to 8.73%, flour 8-MW by 0.25% to 6.35%, and bread volume value by 33.77 mL to 246.50 mL, in comparing it with Chinese Spring. The GMP content and lactic acid SRC showed significant positive correlations with flour processing quality and might be used as indicators for wheat quality. The results were expected to provide a novel route for improving processing quality in wheat quality breeding.

## 1. Introduction

As the second-largest cereal crop in the world, wheat (*Triticum aestivum* L.) is an important source of food, fulfilling the dietary requirements of at least 70% of the global population. It serves as a key ingredient in the preparation of a wide range of food products, including bread, noodles, pastries, biscuits, cookies etc. The total amount of gluten and gliadin proteins in wheat endosperm accounts for approximately 85−90% of the total endosperm protein [1]. Their concentration, composition, and proportions have a crucial impact on gluten, dough function, and end-use products [2]. Wheat glutenin proteins are divided into high-molecular-weight wheat glutenin subunits (HMW-GS) and low-molecular-weight wheat glutenin subunits (LMW-GS), which comprise 7.5% and 32.5% of the total endosperm protein, respectively [3]. Despite their low concentration in wheat grain, HMW-GS are recognized to critically affect the quality of wheat flour processing [4,5].

In wheat, HMW-GS are encoded by the *Glu-1* loci on the long arm of the first homologous chromosome [6]. Each locus contains two closely linked genes, encoding the x-type high-molecular-weight subunit and the y-type low-molecular-weight subunit, respectively [7,8]. It was shown that different allelic variants of the *HMW-GS* gene in wheat were significantly associated with wheat quality [8]. The subunits 1Ax1 or 1Ax2* at the *Glu-A1* locus, 1Bx13 + 1By16, 1Bx17 + 1By18, 1Bx7 + 1By8, and 1Bx7 + 1By9 at the *Glu-B1* locus, and 1Dx5+1Dy10 at the *Glu-D1* locus were significantly and positively correlated with gluten viscoelasticity and strong dough properties, facilitating the formation of stronger gluten to improve the bread processing quality [9,10], whereas, null at the *Glu-A1* locus, 1Bx6 + 1By8 and 1Bx20 + 1By20 at the *Glu-B1* locus, and 1Dx2 + 1Dy12 at the *Glu-D1* locus were associated with poor quality [11,12,13]. As the number of *HMW-GS* alleles is limited in wheat, identifying novel glutenin genes from related species and incorporating them to wheat could provide more genetic resources for improving wheat quality.

A number of *HMW-GS*-particular genes have been previously reported in the germplasm of wheat relatives. For example, Wan et al. [14] cloned two new subunits of the *Glu-C1* and *Glu-D1* loci, 1Cy and 1Dy, respectively, from *Aegilops cylindrica* (genomes C and D). Liu et al. [15] found that the lUx and lUy subunits in *Aegilops umbellulata* (2n = 14, UU) have a high similarity in origin and evolution as the HMW-GS encoded by the D genome and may have potential applications in improving the processing characteristics of hexaploid wheat varieties. Li et al. [16] isolated a new HMW-GS variant, Glu-1St1, from the diploid *Pseudoroegneria stipifolia* (2n = 14, StSt), providing a new perspective on the evolution of HMW-GS in the wheat family. Roy et al. [17] introduced 1Ay21* expressed in durum wheat into Australian bread wheat and improved bread volume and viscoelasticity. Furthermore, Wang et al. [18] suggested that 1Dx3t and 1Dx4t from *Aegilops tauschii* could improve the processing quality of wheat.

*Thinopyrum elongatum* (Host) Nevski is a perennial herbaceous plant possessing several desirable traits, including abundant growth, high grain protein content, tolerance to drought, cold and salinity, and resistance to strip rust, leaf rust, Fusarium head blight, powdery mildew, and other diseases. As it is an excellent genetic resource, and because its E genome is closely related to the wheat genome, which makes it easier to cross with wheat to obtain fertile offspring, it has been used for genetic improvement in wheat through distant hybridization [19,20,21]. Breeders have developed a series of wheat-*Th. elongatum* double diploids using hybridization between decaploid *Th. elongatum* and wheat, such as Agrotana, OK7211542, PWM706, ORRPX, PWM III, PWM 209, and BE-1. Among those, BE-1 has high grain protein content and is resistant to wheat leaf rust and powdery mildew [22,23,24]. Gao et al. [25] analyzed *HMW-GS* genes in the progeny of wheat-decaploid *Th. elongatum* cell fusion and found new subunit types in the hybrid progeny, which could be of potential value for improving wheat quality. In contrast, little research has been conducted on the implementation of tetraploid and diploid *Th. elongatum* HMW-GS for the genetic improvement of wheat.

With the rapid development of molecular biology, the types of molecular marker are increasingly rich as the technology is becoming more mature. The combination of conventional selection and marker-assisted selection not only greatly improve the efficiency of the selection of the target traits, but also accelerate the process of cross-breeding. For example, SSR (simple sequence repeats) primers RM10313 and RM6100 are closely linked to the rice recovery genes *Rf3* and *Rf4* and can be effectively used in the selection of restorer lines containing *Rf3* and *Rf4* genes [26]. Zhang et al. [27] developed dCAPS (derived cleaved amplified polymorphic sequence) molecular markers, RZ2F1/R and RZ2F2/R, based on two restriction endonuclide sites, Hinf Ι and Sty Ι, and Song et al. [28] developed the dCAPS molecular marker DCAPS-172 based on the restriction endonuclide sites of Rsa Ι. Currently, these markers are widely used in the breeding of rice with the thermosensitive male-sterile (*tms5*) gene. In wheat quality breeding, many molecular markers have been developed for HMW-GS, which can effectively distinguish the alleles of *1Ax*, *1Bx*, and *1Dx* [29,30,31]; some of these markers have been used in quality breeding practice and the identification of high generation lines [32]. However, up to now, the effects of HMW-GS of *Th. elongatum* on wheat processing quality were studied in several stages. Moreover, the combination of molecular marker research and breeding practice is not close enough, and there are few reports on the use of HMW-GS of *Th. elongatum* in wheat breeding. So, it is urgent for molecular markers to assist identification in actual breeding populations, so as to improve the efficiency of wheat quality breeding.

To identify novel germplasm for quality breeding in wheat, we cloned an HMW-GS gene, developed specific molecular markers, and measured the contents of grain protein, wet gluten, glutenin macropolymer (GMP), and dough rheological properties, as well as solvent retention capacity (SRC), of the Chinese Spring (CS)-diploid *Th. elongatum* substitution and addition lines.

## 2. Results

### 2.1. Analysis of the Relative Mobility of Th. elongatum HMW-GS

**Figure 1:**
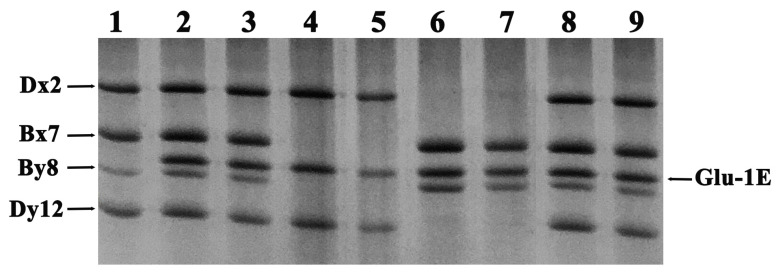
SDS-PAGE detection of HMW-GS in CS-*Th. elongatum* 1E disomic substitution and addition lines. Glu-1E locus-specific subunit of *Th. elongatum*, indicated by arrowhead, is present in substitution and addition lines. 1: Chinese Spring; 2–3: DS1E/1A; 4–5: DS1E/1B; 6–7: DS1E/1D; 8–9: DA1E.

To investigate the types of HMW-GS in the CS-*Th. elongatum* lines, we analyzed the HMW-GS bands of CS, CS-*Th. elongatum* chromosome 1E addition line (DA1E), and substitution lines (DS1E/1A, DS1E/1B, DS1E/1D) by SDS-PAGE electrophoresis. As shown in Figure 1, the four bands in CS, from top to bottom, were identified as subunits 1Dx2, 1Bx7, 1By8, and 1Dy12. In comparing with Chinese Spring, a new band of HMW-GS appeared in the addition line: DA1E. The new band was between 1Bx7 and 1By8. Further analysis showed that the same bands appeared in the substitution lines, DS1E/1A, and the addition line, DA1E, since the 1A locus does not encode glutenin subunits in CS. As expected, the new band appeared in DS1E/1B, and DS1E/1D, whereas 1Bx7 and 1By8 subunits were not showed in DS1E/1B, and 1Dx2 + 1Dy12 subunits were not showed in DS1E/1D. Therefore, the new band was an HMW-GS from the 1E chromosome of *Th. elongatum.*

### 2.2. Cloning and Analysis of the Th. elongatum HMW-GS Gene

**Figure 2:**
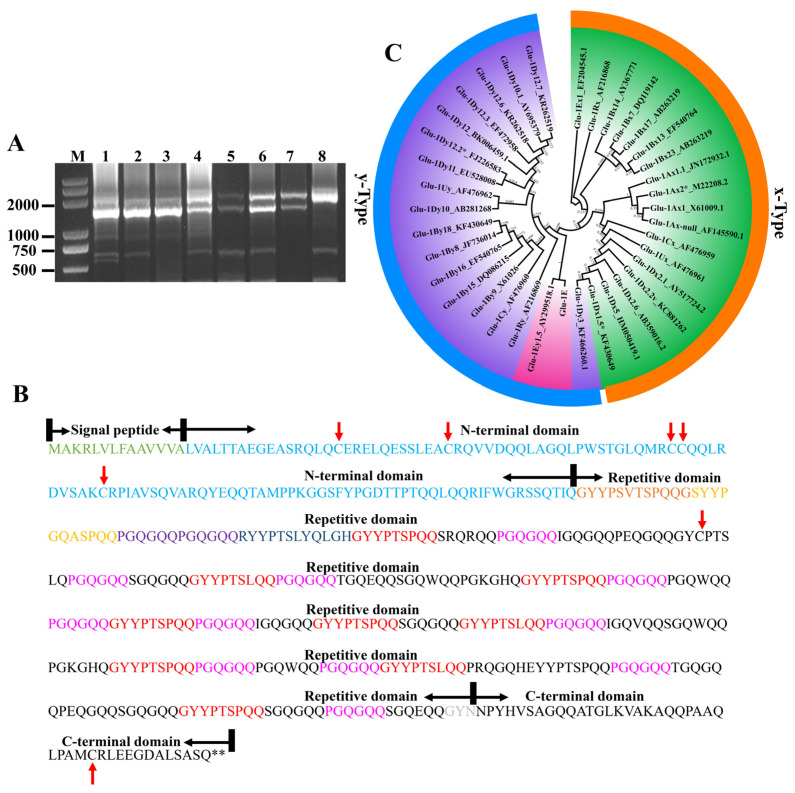
Cloning and analysis of the *Th. elongatum* HMW-GS gene. (**A**) Electrophoresis patterns of P1 and P2 primers; M: Marker; 1: DS1E/1A; 2: DS1E/1B; 3: DS1E/1D; 4: DA1E; 5: diploid *Th. elongatum* (WZ 218); 6: diploid *Th. elongatum* (PI 531717); 7: diploid *Th. elongatum* (PI 531718); 8: CS. (**B**) Amino acid sequence analysis of Glu-1Ey, signal peptide (green); N-terminal domain (light blue); hexapeptide (pink); nonapeptides (red); C-terminal domain (black); cysteine residues (red arrow). (**C**) The tree topologies generated by maximum-parsimony (MP) analyses, derived from HMW-GS amino acid sequence data, conducted using heuristic search with TBR branch swapping.

The *HMW-GS* gene was amplified from the genomic DNA of the *Th. elongatum* chromosome 1E addition and substitution lines of CS, using the degenerative primers P1 and P2. Compared with the bands amplified from CS, the CS-*Th. elongatum* chromosome 1E addition and substitution lines presented an extra band of approximately 1500 bp (Figure 2A). Sequencing of this band showed that the length of gene sequence was 1512 bp, beginning from the start codon, ATG, and ending with two consecutive stop codons, TGATAG without introns (Supplementary Figure S1). Within the sequence, a total of 502 amino acid residues are encoded, including a signal peptide starting with 21 amino acid residues, followed by an N-terminal conserved region consisting of 105 amino acids, a central repeat region composed of hexapeptide (PGQGQQ) and nonapeptide (GYYPTSLQQ) units, and a C-terminal conserved region consisting of 42 amino acids (Figure 2B) at the end. The central repeat region starts with a dodecapeptide (GYYPSVTSPQQG) and an undecapeptide (SYYPGQASPQQ), followed by the intermediate repeating units of hexapeptides and nonapeptides, and ends with a tripeptide (GYN). Besides the consistent sequence PGQGQQ, the hexapeptide also contains repeat units, such as PGKWQE, SGQEKQ, IGKGKQ, PAQGQQ, QGQGQQ, IGQGQQ, PEQGQQ, PGQWQQ, LGQGQQ, SGQGQQ, and others. In addition to GYYPTSLQQ, many other types of repeat units, including GHYPASQQQ, GQIPASQQQ, GHYLASQQQ, and GYYPTSPQQ, are present in the nonapeptide. According to further analysis, the number of major occurrences of β-turning angle QPGQ, YPTS, SPQQ, and QQGY in the intermediate repeat sequences are 15, 11, 9, and 8, respectively. The number of glutamine (Q) residues present is 121, with an average molar percentage of 36.22%, and the number of tyrosine (Y) residues present is 29, with an average molar percentage of 8.68%. In addition, there are seven cysteine residues in *Th. elongatum* polymeric gluten, five of which are at positions 31, 43, 65, 66, and 76 in the N-terminus, one of which is at position 209 near the N-terminus, and one of which is at position 490 in the C-terminus. The phylogenetic analysis shows that the HMW gluten Glu-1E obtained in this study is most closely related to Glu-1Ey15 (AY299518.1), as identified through NCBI, and clustered with the y-type subunit of wheat and its relatives (Figure 2C). Therefore, the sequence analysis suggests that the HMWG Glu-1E obtained in this study belongs to the y-type subunit of *Th. elongatum* and was named Glu-1Ey.

### 2.3. Development of a Molecular Marker for Th. elongatum HMW Glu-1Ey

**Figure 3:**
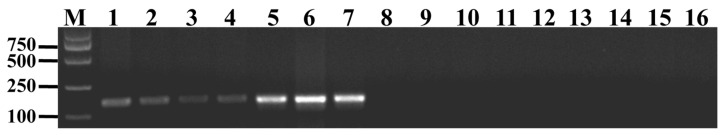
Electrophoresis patterns of molecular marker CMSGlu-1Ey. M: Marker; 1: DS1E/1A; 2: DS1E/1B; 3: DS1E/1D; 4: DA1E; 5: diploid *Th. elongatum* (WZ 218); 6: diploid *Th. elongatum* (PI 531717); 7: diploid *Th. elongatum* (PI 531718); 8: CS; 9: Fielder; 10: NM 1505; 11: TM 1918, 12: YM 9; 13: YM 13; 14: YM 18; 15: ZM 12; 16: SN 23.

In general, an alien chromosome fragment could be detected by genome in situ hybridization or fluorescence in situ hybridization. However, these techniques are time-consuming and laborious, and an alien chromosome cannot be detected when the translocation fragments are small. Specific molecular markers can detect the presence of exogenous genes and can be effectively applied in wheat breeding. In this study, we designed specific primers to detect the specific HMW-GS of *Th. elongatum* by comparing the DNA sequences between *Glu-1Ey* and other *HMW-GS* in wheat. The primer CMSGlu-1Ey was found to amplify the specific bands in the germplasm carried chromosome 1E of *Th. elongatum*, but not those of the different subunits found in common wheat (Figure 3, Table 1). Indeed, the amplified specific bands were confirmed as partial sequences of Glu-1Ey, after having been recovered, sequenced, and compared with *Glu-1Ey*. Therefore, CMSGlu-1Ey can be used as a specific primer for the HMW gluten gene *Glu-1Ey* of *Th. elongatum* in future studies aimed at producing small fragment translocation lines.

### 2.4. Influence of Th. elongatum 1E Chromosome on Grain Quality

Wheat HMW-GS content correlates to wheat processing quality, gluten quality, and gluten strength. Inbred wheat germplasm contains more HMW-GS variant types, which have improved wheat processing quality. In this study, the wheat lines with substitution and addition of *Th. elongatum* chromosome 1E were employed to investigate the effect of diploid chromosome 1E on wheat quality. The wheat containing diploid *Th. elongatum* 1E chromosome showed significantly higher grain protein and wet gluten than CS, with the DS1E/1D line having the highest content of all these components. However, in terms of storage protein components, GMP content was lowest in the DS1E/1D line, at 34.56%, and highest in the DS1E/1A line, at 48.93%. The ratio of wheat glutenin to gliadin (Glu/Gli) was 1.26 in the DS1E/1A line, significantly higher than that of lines DS1E/1B, DS1E/1D, and DA1E. In addition, introducing chromosome 1E considerably enhanced grain hardness, whereas substituting chromosomes from the original homologous chromosomes of wheat with chromosome 1E reduced the hardness to varying degrees (Table 2).

### 2.5. Effect of Th. elongatum Chromosome 1E on Flour Processing Quality

We analyzed the effect of *Th. elongatum* chromosome 1E on the dough rheological properties and flour processing quality using a National Mixograph kneader. The results showed that the introduction of the *Th. elongatum* chromosome 1E had a significant effect on the refining quality of wheat flour. For example, the substitution line DS1E/1A exhibited superior performance when compared to CS and other lines, as evidenced by its mix time (MT), peak height (PH), peak width (PW), 8-min width (8-MW), and bread volume values of 3.71 min, 59.42%, 34.20%, 10.85%, and 840.30 mL, respectively. In contrast, the substitution line DS1E/1D had the lowest values of all the lines, including an MT of 2.30 min and a PW of 15.46%, which are both lower than those of CS. Therefore, regarding the total bread score, the addition line DS1E/1A had the highest score of 81.5, while the addition line DS1E/1D scored only 62.5, when compared to the control CS score of 52.00 (Table 3).

### 2.6. Effect of Th. elongatum Chromosome 1E on Solvent Retention Capacity (SRC)

SRC is the ability of wheat flour to retain solvent under specific centrifugal forces. In addition, it is closely related to wheat quality and is one of the key indicators when evaluating soft wheat. We measured the SRC of the CS-*Th. elongatum* chromosome 1E addition and substitution lines, revealing that the water, sodium carbonate, and sucrose SRCs of the substitution lines DS1E/1A and DS1E/1B were comparable to those of CS, but significantly lower than those of the substitution line DS1E/1D and addition line DA1E. Additionally, the lactic acid SRC of the substitution line DS1E/1D was the lowest of all the lines at only 70.28%, similar to that of CS. In contrast, the lactic acid SRC of the substitution line DS1E/1A was significantly higher than that of the other lines, reaching 107.03% (Table 4).

### 2.7. Correlational Analysis of Quality Traits of CS-Th. elongatum Chromosome 1E Substitution and Addition Lines

**Figure 4:**
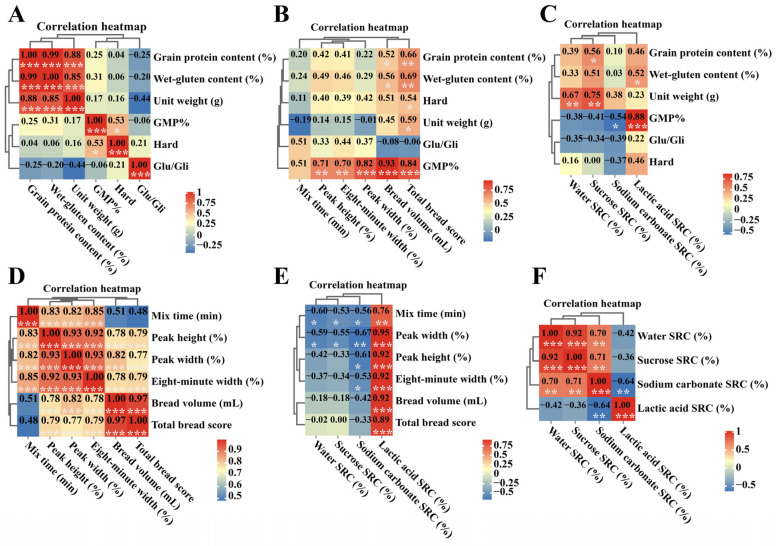
Correlational analysis of quality traits of CS-*Th. elongatum* chromosome 1E substitution and addition lines. (**A**) Correlation analysis of grain quality traits. (**B**) Correlation analysis between grain quality traits and flour processing quality. (**C**) Correlation analysis between grain quality traits and SRC. (**D**) Correlation analysis of flour processing quality. (**E**) Correlation analysis between flour processing quality and SCR. (**F**) Correlation analysis of SRC. *: Correlation is significance at *p* < 0.05 level, **: Correlation is significance at *p* < 0.01 level, ***: Correlation is significance at *p* < 0.001 level.

Pearson’s bivariate correlation analysis was conducted to evaluate the quality traits of the *Th. elongatum* chromosome 1E substitution and addition lines of common wheat. In terms of grain quality, the results shown in Figure 1 indicate a significant, positive correlation between protein content, wet gluten content, and unit weight (R > 0.8, *p* < 0.001), and a weak, positive correlation between GMP and grain hardness (R = 0.53, *p* < 0.05) (Figure 4A). In addition, GMP had a significant, positive correlation with PH and 8-MW (R > 0.7, *p* < 0.01), and a very significant, positive correlation with PW, bread volume, total bread score, and lactic acid SRC (R > 0.8, *p* < 0.001) (Figure 4B,C). However, GMP had a weak, negative correlation with sodium carbonate SRC (R = −0.5, *p* < 0.05) (Figure 4C). For flour processing quality, PH, 8-MW, PW, bread volume, and total bread score, all showed significant positive correlations with each other (R > 0.8, *p* < 0.001) (Figure 4D), as well as with lactic acid SRC (R > 0.7, *p* < 0.01). However, only MT and PW showed weakly negative correlations with water, sucrose, and sodium carbonate SRCs (R < −0.5, *p* < 0.05) (Figure 4E). In addition, within SRCs, apart from a significant, weak, negative correlation (R = −0.64, *p* < 0.01) between the sodium carbonate and lactic acid SRCs, all others showed a significant, positive correlation (R > 0.7, *p* < 0.01) (Figure 4F).

## 3. Discussion

HMW-GS consist of various subunits, which individually and cooperatively affect the processing quality of wheat. Isolation and characterization of the HMW subunit genes in bread wheat and its wild relatives provide new ideas for in-depth research on how to improve wheat quality. All HMW-GS have similar primary structures, typically consisting of three distinct structural domains: The N-terminal region, the C-terminal region, and the repeat region, located in the middle of the peptide chain. A 63-bp sequence, downstream of the start codon, ATG, encodes a signal peptide consisting of 21 amino acids, while two closely linked stop codons, TGATAG, are located at the end of the C-terminus [33]. The HMW-GS sequence obtained from *Th. elongatum* in this study is entirely consistent with that of other common HMW-GS, indicated by the central repeat region consisting of hexapeptide (PGQGQQ) and nonapeptide (GYYPTSLQQ) units with no tripeptide (GQQ) repeat module (Figure 2B). The phylogenetic analysis shows such sequences clustered with the y-type subunit (Figure 2C), demonstrating the y-type subunit gene of *Th. elongatum HMW-GS*. According to the literature, the amino acids in the middle repeat region form a reverse β-fold. Based on different amino acid lengths and compositions of the subunits, the formed β-folds can have varying effects on the dough strength and viscoelasticity [34]. The number of β-fold-forming repeats QPGQ, YPTS, SPQQ, QQGY, as well as the average number and molar percentages of glutamine (Q) and tyrosine (Y) in the amino acid sequence of Glu-1Ey obtained in this study, are all significantly higher than those found in the common wheat. The cross-linking of tyrosine (Y) may also play a role in the structure and functionality of wheat glutenin. While the percentage concentration of tyrosine (Y) may be linked to the significantly higher dough-processing quality of the *Th. elongatum* chromosome 1E substitution and addition lines compared to CS, further investigation is required. In addition, cysteine, a particular amino acid found in HMW-GS, contains sulfur elements that construct the disulfide bonds necessary to form GMP. In contrast to the usual y-type subunit of six cysteine residues, some high-quality subunits, such as 1By9 and 1Dy10, have an extra cysteine residue in the central repeat region near the C-terminal region [33]. This excess cysteine residue may be involved in forming intermolecular disulfide bonds during the formation of GMP, which further improves the viscoelasticity of the dough [35]. Thus, the quantity and distribution of cysteine residues are strongly related to the number of disulfide bonds in the dough, resulting in the formation of intermolecular crosslinks by inter- and intra-chain disulfide bonds. This, in turn, affects the viscoelasticity of the dough [36]. The Glu-1Ey subunit obtained in this study contains seven cysteine residues, of which five are located at positions 31, 43, 65, 66, and 76 in the N-terminus, one at position 209, close to the N-terminus, and one at position 490 in the C-terminus. Consequently, the Glu-1Ey subunit shows potential value for wheat breeding because of its advantages in disulfide bond formation and dough elasticity.

Over the past few decades, some molecular markers, such as simple sequence repeats (SSR), restriction fragment length polymorphism (RFLP), amplified fragment length (AFLP), random amplified polymorphic DNA (RAPD), and diversity arrays technology (DArT), have been used to construct the molecular marker maps to identify QTL associated with important traits [37]. However, because these genetic markers have a certain distance from the target genes, their predictive value depends on the degree of linkage between the markers and the target genes in a specific population. With the increase of reproductive algebra and genetic distance, the probability of breaking the linkage relationship will gradually increase, and the accuracy of molecular marker-assisted selection will decrease. In contrast, functional markers formed by developing functional gene sequences associated with phenotypes have become a hot spot in DNA molecular markers, which are dominant markers and ideal markers for marker-assisted selection in molecular breeding. For example, Liu et al. [38] cloned the *SAI* gene (*SAI-1*) from sweet sorghum, and developed molecular markers for *SAI* molecular research and development of new varieties with high sugar in sweet sorghum through allelic variation analysis. Azmach et al. [39] developed functional markers of CrTRB1-5’TE and CRTRB1-3’TE using three polymorphic loci of the *crtRB1* gene, which encodes β-carotene hydroxylase, to accelerate the breeding efficiency of maize varieties with high carotene. Wei et al. [40] developed a pair of dominant functional markers, POD-3A1 and POD-3A2, based on two allelic variants of the *TaPod-A1* gene (*TaPod-A1a* and *TaPod-A1b*). Pod-3a1 can expand bands in materials containing *TaPod-A1a*, which is associated with low POD activity, and POD-3A2 can expand bands in materials containing *TaPodA1b* type, which is associated with high POD activity. Therefore, functional markers POD-3A1 and POD3A2 can be effectively used in the genetic improvement of POD activity in wheat. Chang et al. [41] developed three molecular markers based on three polymorphic loci in the *TaSAP1-A1* promoter region, namely T7AM5, T7AM2606, and T7AM39. Association analysis showed that the three molecular markers were significantly associated with agronomic traits such as spike length, peduncle length, spikelet number per spike, grain number per spike, and 1000-grain weight. It can be seen that molecular marker technology has the advantages of simplicity, economy, speed, and high efficiency, and is the preferred means to speed up the process of crop breeding and to improve breeding efficiency. However, compared with many traits, the number of molecular markers for identifying wheat *HMW-GS* genes is still very little, and only a few subunits can be accurately and quickly identified. In this study, we developed a dominant molecular marker, CMSGlu-1Ey, using cloned y-type *HMW-GS* gene sequence of *Th. elongatum*, which can effectively track and detect the y-type *HMW-GS* gene of *Th. elongatum* under the wheat background. It will play a great role in improving the quality of wheat by using HMW-GS of *Th. elongatum*.

Additionally, quality traits are one of the critical factors involved in the selection and breeding of high-quality wheat varieties. The presence of HMW-GS is strongly related to the processing quality of wheat flour and plays a unique and vital role in gluten characteristics. A total deficiency of HMW-GS has been shown to considerably reduce gluten strength, PH, PW, tensile resistance, and other parameters of wheat [42]. Furthermore, the simultaneous deletion of *Glu-1A* and *Glu-1D* increased the diameter, crispness, and height of resulting cookies [43]. However, of the three, *Glu-lA*, *Glu-1B*, and *Glu-1D* loci, the deletion of the *Glu-1D* locus has the worst negative impact on dough quality with regards to strength and elasticity [44]. It has been reported that all species in the wheat family have HMW-GS. Despite the common wheat’s low genetic diversity, the HMW-GS of close wheat relatives are highly varied, offering valuable genetic resources for the improvement of wheat quality [45]. For example, 45 *HMW-GS* gene haplotypes have been identified in *Aegilops tauschii* to date, of which the subunits 1Dx3t and 1Dx4t can improve the processing quality of wheat [46,47]. In addition, *Glu-1U*-encoded HMW-GS can also improve wheat quality. For example, *Glu-1U^g^* of *Ae. geniculata* significantly improved the rheological properties of dough [48]; *Glu-1U^u^* of *Ae. umellulata* increased the GMP content [49,50]; *Glu-1U^b^* of *Ae. biuncialis* increased the protein content, Zeleny sedimentation, grain hardness, and wet gluten content [51]; and *Glu-1U^k^* from *Ae. kotschyi* increased SDS sedimentation in wheat [52]. Furthermore, *Dasypyrum villosum* HMW-GS *Glu-1V* can improve protein content, Zeleny sedimentation, wet gluten content, and rheological properties of small fragment translocation line NAU425, which shows potential in wheat quality improvement [53]. In the present study, we analyzed the effect of *Th. elongatum* chromosome 1E on wheat quality and found that its presence significantly raised the protein, wet gluten, hardness, and bulk density of the grain. In terms of bread processing quality, the addition line, DA1E, and substitution lines, DS1E/1A and DS1E/1A, were superior in quality to the substitution lines, DS1E/1D, and control, CS. Indeed, in substitution line DS1E/1D, the substitution of the *Th. elongatum* HMW-GS in DS1E/1D by the high-quality subunit 2 + 12 in CS may cause a reduction in quality. Previous studies have found a significant correlation between GMP content and wheat flour processing quality. In the present study, we also found that the HMW-GS of the DS1E/1D line strongly increased the grain GMP content and was positively correlated with bread volume and overall bread score (R = 0.93 and 0.84, respectively; Figure 1B). Wheat flour lactic acid SRC reflects its gluten properties and are often positively correlated with gluten strength, with higher values indicating better gluten properties in soft wheat. Here, we found that *Th. elongatum* HMW-GS remarkably increased the lactic acid SRC and had significant, positive correlations with the GMP, MT, PH, PW, 8-MW, bread volume, and overall bread score (Figure 4C,E). Therefore, to improve wheat quality using *Th. elongatum* HMW-GS, the GMP and lactic acid SRC content can be employed to select the lines with excellent bread-processing quality for future wheat breeding.

The HMW-GS carried by *Th. elongatum* was different from that of common wheat. Compared with the control Chinese Spring, the high-molecular-weight glutenin subunits of *Th. elongatum* significantly increased grain protein content by 3.76% to 5.11%, wet-gluten content by 6.55% to 8.73%, and unit weight by 25.88 g to 40.75 g; further, it increased flour 8-MW by 0.25% to 6.35% and bread volume value by 33.77 mL to 246.50 mL. Moreover, the solvent retention capacity of lactic acid and sucrose was increased by more than 19% and 14%, respectively, thereby contributing remarkably to overall dough quality. Therefore, in future studies, the HMW-GS of *Th. elongatum* can be studied in wheat with various genetic backgrounds via small fragment translocation lines to determine the effects on wheat and flour processing quality. In conclusion, intermediate materials can be produced for wheat breeding and can provide germplasm resources for wheat quality improvement.

## 4. Materials and Methods

### 4.1. Plant Materials

The four varieties of *Th. elongatum* and CS used in this study were planted in the Liuhe wheat-planting trial base of Jiangsu Academy of Agricultural Sciences (Nanjing, Jiangsu, China). The field was planted in sequential arrangement, with a row length of 1.5 m and row spacing of 20 cm, using three groups of replications and manually sowing approximately 30 seeds in each row. The fertility of the trial field was even and moderate under the local conventional field management. Two replicates of seeds were harvested, packaged separately, and numbered by location to identify and analyze the subunit composition of wheat glutenin. The grains harvested in the same year from various lines were used for experimental evaluation of quality traits. The harvested grains were fumigated and stored for approximately 1 month before being ground and left at 24 °C for over a month. In addition, three diploid lines *Th. elongatum* (WZ 218, PI 531717, PI 531718) and the common wheat with different high-molecular-weight gluten subunit types (Neimai 1505, Taimai 1918, Yumai 9, Yumai 13, Yumai 18, Zhenmai 12, Shannong 23, Fielder, Chinese Spring) were all planted in the greenhouse at Yangzhou University.

### 4.2. Protein Extraction from Grain and SDS-PAGE

Glutenin extraction from non-embryonic half-wheat grains followed the procedure described by Garg et al. [54], with minor modifications. Briefly, 10 µL of samples were added to a sodium dodecyl-sulfate polyacrylamide gel electrophoresis (SDS-PAGE) gel, consisting of 8% gradient separation gel (pH 8.5) and 4% stacking gel (pH 6.8), and electrophoresed at a constant current of 10 mA for 10 h. After electrophoresis, the gels were run at a constant current of 0.5 mA. Next, the gels were stained with 0.1% *w*/*v* Komas Brilliant Blue R-250 (A610037, Sangon Biotech, Shanghai, China), 25% *w*/*v* isopropanol (A600918, Sangon Biotech, Shanghai, China) and 10% *w*/*v* acetic acid (A501931, Sangon Biotech, Shanghai, China) for 10 h. The gels were then rinsed several times with deionized water and left submersed until the bands became visible.

### 4.3. Determination of Wheat Quality

The determination method of wheat quality mainly refers to the article by Liu et al [55]. Wheat grain hardness was determined using an SKCS 4100 analyzer (Perten, Zürich, Switzerland), which provides a hardness index of wheat, based on the system-defined formula. Using a near-infrared reflectance (NIR) spectrometer DA7200 (Perten Instruments, Huddinge, Sweden), the protein content, the wet gluten content, GMP%, ratio of wheat glutenin to alcohol-soluble protein (Glu/Gli), and other parameters of the kernels and flour were determined. The grains were dried and milled using a Buhler 202 mill (Germany), according to the AACC26-21A protocol. The mixing time (MT), peak height (PH), peak width (PW), eight-minute width (8-MW), and other mixing parameters were determined using an electronic mixer (National Mixograph, Manhattan, KS, USA), according to the AACC 54-40 method. The bread was prepared using the GB/T 14611-93 method (“Wheat bread baking direct fermentation method”), and were scored by the improved international method. The specific indexes and scoring criteria are shown in Table 5. The SRC parameters were determined according to the AACC56-11 method, and all sample weights and reagent amounts were converted to 1/5 of the standard amount. The significance and correlation analysis of quality parameters were performed using IBM SPSS 25.0, and the graphs were plotted using GraphPad Prism 8.

### 4.4. DNA Isolation and PCR Amplification

Genomic DNA was extracted from wheat leaves by the CTAB method [56]. Degenerative primers P1 and P2 (P1: ATGGCTAAGCGGYTRGTCCTCTTTG, P2: CTATCACTGGCTRGCCGACAATGCG) were used to amplify the high-molecular-weight gluten gene sequence of *Th. elongatum*. PCR amplification was carried out in a 25 μL reaction, containing 1 μL genomic DNA (100 ng/μL), 12.5 μL 2× Phanta Master Mix (Vazyme Biotech Co., Nanjing, China), 1 μL 10 μmol/L of each primer, and 9.5 μL double-distilled water. The PCR procedure was as follows: 94 °C for 5 min, followed by 35 cycles of 94 °C for 45 s, appropriate anneal temperature (65 °C) for 45 s, 72 °C for 2 min, and a final extension for 10 min at 72 °C. Amplified PCR products were separated on 1% agarose gels at 130 V for 20 min, stained with ethidium bromide, and visualized using ultraviolet (UV) light. The amplified DNA product was purified by the Universal DNA Purification Kit (Tiangen Biotech Co., Ltd., Beijing, China) and cloned by the 5-min TA/Blunt-Zero Cloning Kit (Vazyme Biotech Co., Nanjing, China).

### 4.5. Phylogenetic Analyses

ClustalX in MEGA6 (6.06) was used for multiple sequence alignments with the default parameters. Multiple sequence alignments were adjusted manually to minimize gaps [57]. Multiple sequences from each accession were first compared to identify distinct copies of sequences by ClustalX. The high-molecular-weight glutenin subunit sequences of wheat and its related species were downloaded from the NCBI website for phylogenetic analysis (Supplementary Table S1). Phylogenetic analysis, using the maximum-parsimony (MP) method, was performed with the computer software MEGA6 (6.06). The MP trees were constructed by performing a heuristic search using the Tree Bisection-Reconnection (TBR), with Mul-Trees on, and 10 replications of random addition sequences, with the stepwise addition option. Overall character congruence was estimated by the consistency index (CI) and the retention index (RI). To test the robustness of clades, bootstrap (BS) values with 1000 replications were calculated by performing a heuristic search, using the TBR option with Mul-Tree on [58].

## Figures and Tables

**Table 1:** The common wheat with different high-molecular-weight gluten subunit types.

Wheat Variety	Abbreviation	Subunit Composition
Neimai 1505	NM 1505	Ax1, Dx5 + Dy10, Bx13 + By16,
Taimai 1918	TM 1918	N, Dx5 + Dy10, Bx17 + By18
Yumai 9	YM 9	Ax2*, Dx5 + Dy10, Bx7 + By8
Yumai 13	YM 13	Ax2*, Dx2 + Dy12, Bx7 + By8
Yumai 18	YM 18	Ax2*, Dx5 + Dy10, Bx7 + By9
Zhenmai 12	ZM 12	Dx5 + Dy10, Bx7 + By9
Shannong 23	SN 23	Ax1, Dx5 + Dy10, Bx6 + By8
Fielder	Fielder	Dx2 + Dy12, Bx14 + By15
Chinese Spring	CS	Dx2 + Dy12, Bx7 + By8

**Table 2:** Analysis of grain quality trait of CS-*Th. elongatum* 1E chromosome addition and substitution lines.

Materials	Grain Protein Content (%)	Wet-Gluten Content (%)	GMP (%)	Glu/Gli	Hard	Unit Weight (g)
DS1E/1A	17.92 ± 0.36 b	34.51 ± 0.51 a	49.23 ± 1.88 a	1.23 ± 0.23 a	47.83 ± 0.15 b	925.54 ± 1.32 b
DS1E/1B	17.16 ± 0.17 c	32.96 ± 0.85 b	45.94 ± 0.96 b	0.90 ± 0.02 c	44.37 ± 0.12 e	928.84 ± 2.64 b
DS1E/1D	18.37 ± 0.20 a	34.56 ± 0.41 a	32.41 ± 0.73 d	0.99 ± 0.11 b	45.33 ± 0.27 d	940.41 ± 4.76 a
DA1E	17.02 ± 0.13 c	32.38 ± 0.70 b	48.73 ± 0.54 a	0.91 ± 0.06 c	49.03 ± 0.39 a	939.11 ± 1.73 a
CS	13.26 ± 0.22 d	25.83 ± 0.25 c	36.70 ± 2.31 c	1.15 ± 0.02 ab	46.15 ± 0.21 c	899.66 ± 4.78 c

Note: Data are shown in mean ± standard deviation (SD), and different letters for each column show significance at *p* < 0.05.

**Table 3:** Determination of flour processing quality parameters for the CS-*Th. elongatum* chromosome 1E addition and substitution lines.

Materials	Mix Time (min)	Peak Height (%)	Peak Width (%)	8-min Width (%)	Bread Volume (mL)	Total Bread Score
DS1E/1A	3.71 ± 0.05 a	59.55 ± 1.39 a	34.14 ± 0.64 a	10.85 ± 1.70 a	840.21 ± 5.17 a	81.50 ± 0.72 a
DS1E/1B	2.92 ± 0.66 b	51.64 ± 1.96 b	25.58 ± 1.61 b	7.15 ± 1.29 b	783.18 ± 3.12 c	72.11 ± 1.26 b
DS1E/1D	2.30 ± 0.13 c	47.46 ± 0.09 c	15.48 ± 1.52 d	4.75 ± 0.77 cd	627.48 ± 7.94 d	62.36 ± 1.84 c
DA1E	2.35 ± 0.16 bc	50.88 ± 2.48 b	24.03 ± 0.53 b	6.65 ± 1.07 bc	823.21 ± 7.57 b	79.01 ± 1.41 a
CS	2.55 ± 0.23 b	47.01 ± 2.09 c	19.89 ± 1.98 c	4.50 ± 0.30 d	593.71 ± 8.69 e	52.44 ± 2.22 d

Note: Data are shown in mean ± standard deviation (SD), and different letters for each column show significance at *p* < 0.05.

**Table 4:** Determination of the SRCs of CS-*Th. elongatum* chromosome 1E addition and substitution lines.

Materials	Water SRC (%)	Sodium Carbonate SRC (%)	Lactic Acid SRC (%)	Sucrose SRC (%)
DS1E/1A	58.20 ± 0.63 b	69.72 ± 0.15 c	107.37 ± 1.37 a	107.82 ± 2.10 c
DS1E/1B	58.76 ± 0.50 b	72.62 ± 0.95 ab	88.48 ± 1.23 b	109.63 ± 1.92 c
DS1E/1D	63.24 ± 0.70 a	73.94 ± 2.01 a	70.29 ± 0.34 c	131.74 ± 7.49 a
DA1E	62.24 ± 0.87 a	72.38 ± 0.21 ab	88.74 ± 0.17 b	120.04 ± 1.74 b
CS	58.72 ± 0.32 b	71.85 ± 0.52 b	69.43 ± 1.73 d	105.42 ± 1.67 c

Note: Data are shown in mean ± standard deviation (SD), and different letters for each column show significance at *p* < 0.05.

**Table 5:** Scoring criteria for bread evaluation.

Appearance (Out of 10)	Internal Structure (Out of 20)	Elasticity (Out of 20)	Taste (Out of 10)	Volume/mL (Out of 40)
Rounded top and upright rising, no scorched skin (8–10)	Bright white, very uniform, rectangular, very thin cell walls (17–20)	Very soft, high elasticity, recovers very quickly after pressing (17–20)	Fine taste, slightly sweet, yeasty and salty flavor, very delicate and resilient (8–10)	≥850 (34–40)
Medium top and rising, slightly scorched skin (7)	Slightly creamy, uniform cells, thin cell walls (15–16)	Soft, medium elasticity, recovers after pressing (15–16)	No obvious aroma and odor, delicate and resilient (7)	800–849 (30–33)
Flat top, no rising, dark skin (6)	Dark, non-uniform cells, thick cell walls (12–14)	Not soft, poor viscoelasticity, no recovery (12–14)	Rough and coarse taste, crumbs present (6)	750–799 (24–29)

## Data Availability

Data is contained within the article or Supplementary Materials.

## Supplementary Materials

The following supporting information can be downloaded at: https://www.mdpi.com/article/10.3390/ijms241311072/s1.
