# Supplementary material for: Molecular Characterization and Marker Development of the HMW-GS Gene from Thinopyrum elongatum for Improving Wheat Quality
Source: Int J Mol Sci. 2023 Jul 4;24(13):11072. doi: 10.3390/ijms241311072 (PMC10341674; doi:10.3390/ijms241311072)
Supplement: Supplementary file 1 [file ijms-24-11072-s001.zip › Supplementary Figure S1.pdf]

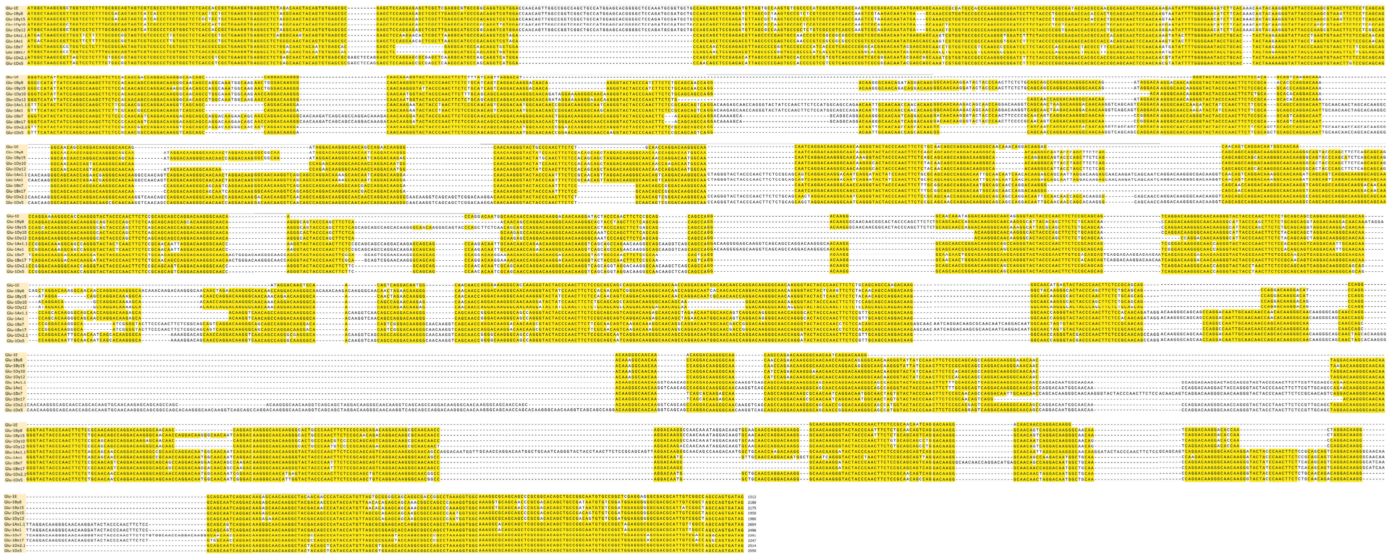

Figure S1. Sequence alignment of high molecular weight gluten genes of different subunits between *Th. elongatum* and common wheat. The yellow highlight indicates that the sequence has high homology.
